# Morphological description and DNA barcoding study of sand rice (*Agriophyllum squarrosum*, Chenopodiaceae) collected in Kazakhstan

**DOI:** 10.1186/s12870-017-1132-1

**Published:** 2017-11-14

**Authors:** Yuliya Genievskaya, Saule Abugalieva, Aibatsha Zhubanysheva, Yerlan Turuspekov

**Affiliations:** 1 Institute of Plant Biology and Biotechnology, Almaty, Kazakhstan 050040; 2 Aktobe breeding station, Aktobe region, Kazakhstan 030014

**Keywords:** *Agriophyllum squarrosum*, *Agriophyllum minus*, Sand rice, Shrub, DNA barcoding, ITS, *mat*K

## Abstract

**Background:**

Sand rice (*Agriophyllum squarrosum* (L.) Moq.) is an annual shrub-like plant adapted to the mobile sand dunes in desert and semi-desert regions of Asia. It has a balanced nutrient composition with relatively high concentration of lipids and proteins, which results in its nutrition being similar to legumes. Sand rice’s proteins contain the full range of essential amino acids. However, calories content is more similar to wheat. These features together with desert stress resistance make sand rice a potential food crop resilient to ongoing climate change. It is also an important fodder crop (on young stages of growth) for cattle in arid regions of Kazakhstan. In our work, sand rice samples were collected from two distant regions of Kazakhstan as a part of the nation-wide project to determine genetic variation of the native flora.

**Results:**

Samples were collected in western and southeastern parts of Kazakhstan separated by distances of up to 1300 km. Sequences of the nuclear ribosomal DNA ITS1-5.8S-ITS2 region and the chloroplast *matK* gene confirmed the identity of species defined by morphological traits. Comparison with GenBank sequences revealed polymorphic sequence positions among Kazakh populations and GenBank references, and suggested a distinction among local populations of sand rice. The phylogenetic analysis of nucleotide sequences showed a clear partition of *A. squarrosum* (L.) Moq. from *Agriophyllum minus* Fisch. & C.A. Mey, which grows in the same sand dunes environment.

**Conclusions:**

DNA barcoding analyses of ITS and *matK* sequences showed a segregation of *A. squarrosum* from *A. minus* into separate clades in Maximum-Likelhood dendrograms. ITS analysis can be successfully used to characterize *A. squarrosum* populations growing quite distant from each other. The data obtained in this work provide the basis for further investigations on *A. squarrosum* population structure and may play a role in the screening of sand rice plants growing in desert and semi-desert environments of Central Asia and China.

## Background

Deserts and semi-deserts occupy more than the half of Kazakhstan’s territory. A very limited number of plants can survive in extreme conditions of xeric, hot sands. One of the examples of successfully growing plants in harsh desert environments is sand rice, *Agriophyllum squarrosum* (L.) Moq. In Kazakhstan and Central Asia it is known as kumarchik (from the Turkic “kum” – sand). It is an annual endemic plant found in desert and semi-desert regions of Asia. *A. squarrosum* belongs to the tribe Corispermeae within the subfamily Chenopodioideae of Chenopodiaceae [1, 2]. *Agriophyllum* contains five species and four of them, including *A. squarrosum*, occur in Kazakhstan [3].

*A. squarrosum* (old name: *A. arenarium* M.Bieb.) is a shrub-like plant 20-100 cm tall. Stems are erect, light green, firm, ribbed, covered with ramified hairs when young, and branched from the base. Leaves are sessile, lanceolate to linear, 1.3-7 cm × 1-10 mm in size. Small flowers form the inflorescence, a spike. Spikes are axillary, sessile, dense, ovoid or ellipsoid in shape. Seeds are subglobose, 1.3-2 mm long, sometimes speckled with light brown two-pointed cornicles. Sand rice flowers from August to October; seeds ripen during the same period. Seeds of *A. squarrosum* are very light, covered in a membranous husk. After ripening, the husk is cracked into two parts and seeds are easily dispersed by wind [3].

Sand rice has a diverse economic importance. Each organ of this plant, from root to seeds, finds its own application in human’s life. Although the plants grow in infertile sand, *A. squarrosum* has a high concentration of nutrients in its seeds and other organs. Since ancient times, nomads had used seeds of sand rice as a food [4]. As it is not possible to grow cereals in the desert on a large scale, shepherd-nomads collected sand rice seeds, ground them and used the flour for flat cakes and porridge [5]. Nutritional properties of sand rice represent a combination of relatively high proteins (23.2% of dry weight) and lipids (9.7% of dry weight) content together with carbohydrates (up to 45% of dry weight). Proteins include the full set of essential amino acids required for human diet [6]. The nutritional properties are similar to legumes, such as soybean or chickpea, but with a lower amount of calories. At the vegetative stage *Agriophyllum* species are heavily used as a pasture fodder plants for sheep and camels [7]. Young fresh stems and leaves of sand rice are suitable for silage. Medical usage of sand rice is also well described in the literature. According to the ancient books in Mongolian medicine, decoction of seeds had been used as antipyretic and analgesic medical [8]. In China, *A. squarrosum* is routinely used for the treatment of kidney inflammation [6].

The root system of sand rice plants has a rather unique structure and includes a long taproot and almost equally long lateral roots near the soil surface. The length of the taproot can be comparable to the height of above ground organs [9, 10]. Some lateral roots can reach a length of 5 m by the time the main stem has grown to just 67 cm [6]. They enable the plant to gain a foothold in sand and withstand strong sandstorms. Therefore, *A. squarrosum* has been cultivated in deserts and semi-deserts of Central Asia for sand stabilization [11, 12].

Physiology and morphology of sand rice are ideally adapted to the extreme desert conditions [13], which make *A. squarrosum* interesting for studying its genomic basis of xeric adaptations. In the last 10 years, there were several works dedicated to the search of candidate genes responsible for the tolerance to heat and drought stresses [14]. One of the current primary goals is the domestication of sand rice in desert environments [15] via development of plants with larger grains and higher yield, and to exclude unfavorable phenotypic traits such as seed shattering, thorns, etc.

A survey of the scientific literature revealed that, at present, no studies have been published on the taxonomy of the small genus *Agriophyllum*. We conducted this study to identify the intraspecific genetic diversity of *A. squarrosum* using barcoding markers and to see how clearly it is diverged from *A. minus*, which also widely grows in desert areas of Kazakhstan, partly in sympatry with *A. sqarrosum*. DNA barcoding is a powerful and efficient tool for the identification of poorly studied species [16–18]. Previous reports suggested that phylogenetic analysis of plants in the Caryophyllales can be effective using major universal DNA markers [19]. In our study here the chloroplast genome marker *mat*K [20] and the nuclear ribosomal DNA region including the internal transcribed spacers 1 and 2 (ITS) together with the 5.8S rRNA gene [21] were utilized. The study is a part of larger nation-wide project [22] for genotyping of endemic, rare, and economically important species of Kazakhstan’s flora that combine efforts of local botanists and geneticists from biotechnology research organizations, botanical gardens, state nature parks and reserves.

## Methods

### Materials sampling and morphological identification

A total of seven populations of *Agriophyllum* species were collected from two distant regions of Kazakhstan (Table 1).

**Table 1:** Materials and sampling locations

Region	Species, population №	Number of collected plants	Coordinates	Elevation, m
Southeastern KZ (Almaty region, Moyynkum Sands)	*A. squarrosum* pop.1	10 plants	44°34′55.1″ N 076°58′30.3″ E	411
	*A. squarrosum* pop.2	5 plants	44°43′24.1″ N 076°44′21.3″ E	409
	*A. squarrosum* pop.3	10 plants	44°38′34.2″ N 076°57′03.5″ E	434
	*A. squarrosum* pop.4	10 plants	44°39′48.1″ N 076°47′25.6″ E	429
	*A. minus* pop.1	5 plants	44°13′27.1″ N 076°58′30.3″ E	463
Western KZ (Aktubinsk region, Bol’shie Barsuki Sands)	*A. squarrosum* pop.5	10 plants	47°51′37″ N 059°52′58″ E	189
	*A. minus* pop.2	5 plants	47°22′62″ N 059°59′28″ E	183

**Fig. 1:**
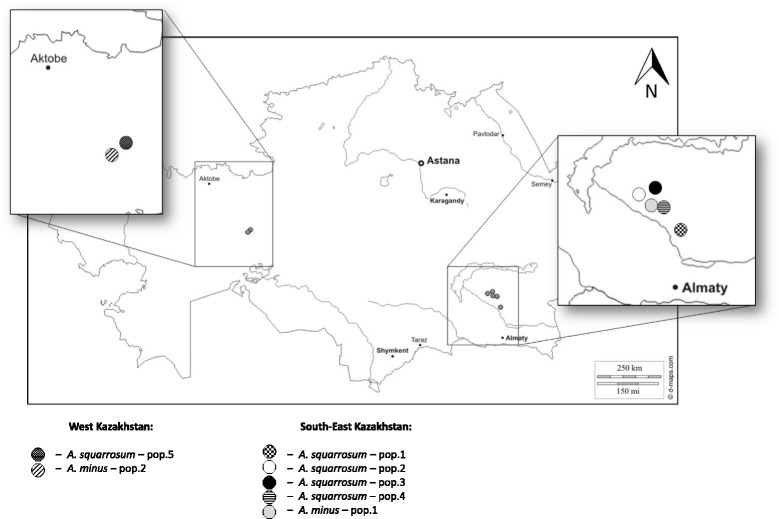
Collection sites of sand rice in Kazakhstan. The geographic positions of the locations on the map are provided in Table 1

The first population of *A. squarrosum* in western Kazakhstan, Aktobe region, Bol’shie Barsuki Sands was collected in 2015. The remaining four populations in southeastern Kazakhstan, Almaty region, Moyynkum Sands were collected in 2016 (Fig. 1).

Both the western and southeastern territories are characterized by extreme desert climate and are usual habitats for sand rice [3, 23]. In order to compare morphology and genetic diversity among populations of two closely related species, two populations of *A. minus* were also collected in the same regions of Kazakhstan. Total distances between the two sampling areas varied from 1250 to1300 km. The approximate distance between each of the populations from one region is 4-6 km. The populations were separated from each other by sand dunes not inhabited by the species. Whole plants and segments of branches were chosen as materials for our analysis. Entire plants were used to make herbarium voucher specimens. Dry branches and seeds were packed and annotated on individual paper bags in order to avoid contamination.

### DNA extraction, amplification and sequencing

Three plants from each population were chosen for molcular analysis. Total genomic DNA was extracted from dry leaf material according to the modified Dellaporta DNA extraction protocol [24]. PCR fragments were amplified for the *maturase* K gene of the chloroplast genome (*mat*K) [25] and the nuclear ribosomal ITS region [26].

All PCR reactions were carried out in 16 μl volumes in a Veriti Thermo cycler (Applied Biosystems, Foster City, CA, USA). One PCR reaction contains 4 mM of each dNTP, 6.4 mM of primer mix, 1.6 U of Taq DNA polymerase and 80 ng of total genomic DNA. Protocols for PCR reactions were taken from Jun et al. [27]. Primers chosen for PCR, their nucleotides sequence and sizes are given in Table 2.

**Table 2:** Primer sequences for the rDNA ITS region and the chloroplast *mat*K gene

Primers	Nucleotide sequence	Annealing temperature	Expected sizes^a^
ITS1nF ITS4nR	5′-AGAAGTCGTAACAAGGTTTCCGTAGG- 3′ 5′-TCCTCCGCTTATTGATATGC- 3′	58 °C	600-680 bp
matK-F matK-R	5′-CCTATCCATCTGGAAATCTTAG- 3′ 5′-GTTCTAGCACAAGAAAGTCG- 3′	50 °C	750-800 bp

^a^expected sizes are approximate lengths of amplicons usually obtained from Caryophyllales according to data in GenBank [34]

PCR products were run in 1.5% agarose gels. Single bands with expected sizes around 750 bp for *mat*K and 650 bp for ITS were cut out from gels and purified using ULTRAPrep® Agarose Gel Extraction Mini Prep Kit (AHN Biotechnologie GmbH, Nordhausen, Germany) according to the protocol provided by the company.

Purified DNA amplicons were used for the sequence reactions with forward and reverse primers separately. All reactions were performed with the BigDye Terminator Cycle Sequencing technology (Applied Biosystems, Foster City, CA, USA). Each reaction was carried out in 20 μl volume according to the protocol of the company.

### Alignment and phylogenetic analyses

DNA sequences were imported to MEGA 6.06 [28] for an alignment together with sequences from the GenBank database with the addition of the outgroup species *Chenopodium opulifolium* Schrad. ex Koch & Ziz. and *Chenopodium quinoa* Willd. Maximum Likelihood analyses [29] with 1000 bootstrap replications and the Tamura-Nei model [30] of sequence evolution were used to construct a phylogenetic tree.

## Results

In the laboratory, each individual plant was initially identified at the species level by taxonomists using descriptions given in The Flora of Kazakhstan [3]. The species identification was based on differences in the structure of seeds, leaves and spikes (Table 3).

**Table 3:** Morphometric analysis of key sand rice traits. All data are represented as a mean value for all plants in each population with SEM

Region	Species, population №	Leaf length, cm	Seed diameter, cm	Spike length, cm	50 seeds weight, mg	Number of seeds per spike
Southeastern KZ	*A.squarrosum* pop.1	4.36 ± 0.06	0.52 ± 0.021	1 ± 0.033	40.8 ± 0.573	16 ± 0.422
	*A.squarrosum* pop.2	3.2 ± 0.041	0.61 ± 0.041	1.6 ± 0.071	60 ± 1.78	20 ± 0.817
	*A.squarrosum* pop.3	8.18 ± 0.105	0.51 ± 0.021	2 ± 0.045	57 ± 0.667	26 ± 0.422
	*A.squarrosum* pop.4	2.7 ± 0.039	0.5 ± 0.021	1 ± 0.021	53 ± 0.869	18 ± 0.422
	*A.minus* pop.1	1.9 ± 0.077	0.51 ± 0.032	1.2 ± 0.055	33 ± 1	14 ± 0.512
Western KZ	*A.squarrosum* pop.5	4 ± 0.052	0.58 ± 0.02	1.4 ± 0.037	50 ± 0.803	18 ± 0.422
	*A.minus* pop.2	1.79 ± 0.062	0.5 ± 0.03	1.7 ± 0.079	32 ± 0.912	12 ± 0.417

Plant measurements showed a clear difference of *A. squarrosum* from *A. minus* using several traits, such as leaf length, weight and number of seeds (*p* < 0.001). The results from a *t-test* indicated that *A. squarrosum* (in comparison to *A. minus*) had higher seed weights (*P* < 0.01), more seeds per spike (*P* < 0.05), and longer leaves (*P* < 0.05).

### Variability at ITS region and *mat*K gene

**Fig. 2:**
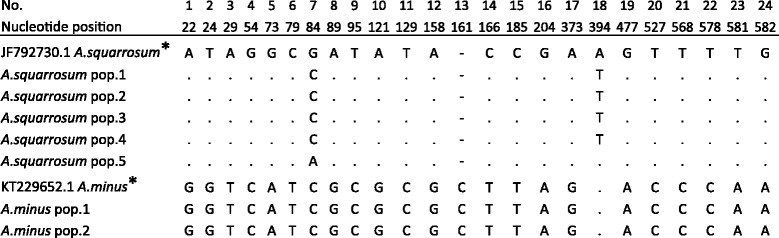
Polymorphic sites of *A. squarrosum* and *A. minus* detected in ITS. *A. squarrosum* pop. 1, 2, 3, 4 and *A. minus* pop. 1 were collected in Southwest Kazakhstan; *A. squarrosum* pop. 5 and *A. minus* pop. 2 – West Kazakhstan. * – indicates GenBank sequences, with reference numbers, from the NCBI database

From each population listed in Table 1 three individual plants were selected for DNA extraction. In total, 21 DNA samples were sequenced for ITS and *mat*K. ITS sequences had a size range from 629 to 662 bp. The sequences were aligned in MEGA 6.06 and compared to GenBank references. Since sequences showed no variability in both ITS and *mat*K among the individuals of single populations, one sample per population was been chosen for further work. As a result, 24 nucleotide substitutions in the ITS region were detected that distinguish *A. minus* from *A. squarrosum* (Fig. 2). The total variability of ITS sequences in this study was 3.6%*.*

We found no variation in ITS sequences among samples from two populations of *A. minus* collected in different parts of Kazakhstan and NCBI GenBank specimens. Sequences of ITS from *A. squarrosum*, on the contrary, showed two polymorphic nucleotides (pos. 84 and 394) distinguishing populations from southeastern and western parts of Kazakhstan (Fig. 2). The same two nucleotides separated our *A. squarrosum* samples from sequences deposited in NCBI GenBank and samples from southeastern populations of *A. squarrosum,* but only one nucleotide in position 394 was different between the GenBank accession (from China) and samples from the West Kazakhstan population (Fig. 2).

**Fig. 3:**
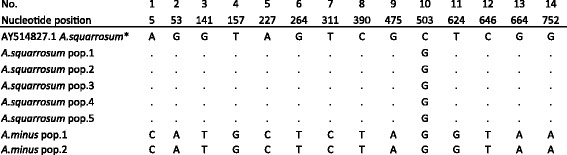
Polymorphic sites of *A. squarrosum* and *A. minus* detected in the *mat*K gene. *A. squarrosum* pop. 1, 2, 3, 4 and *A. minus* pop. 1 were collected in southeastern Kazakhstan; *A. squarrosum* pop. 5 and *A. minus* pop. 2 in western Kazakhstan. * – indicates GenBank sequences, with their reference numbers

Sequences of *mat*K for samples of *A. squarrosum* in both regions were all identical. Fourteen polymorphic sites between *A. squarrosum* and *A. minus* were identified in this study. The nucleotide in position 503 separated *A. squarrosum* growing in Kazakhstan from the sequence available in GenBank (Fig. 3). The total length of the *mat*K alignment in this study was 768 nucleotides. The variability of sequences was 1.8%.

### Differentiation of two *Agriophyllum* species based on ITS region and matK gene sequencing

**Fig. 4:**
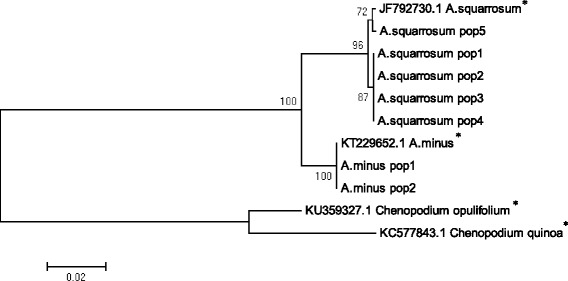
Maximum Likelihood phylogenetic tree resulted from analysis of the ITS sequences. The length of branches is based on Maximum Composite Likelihood analysis and numbers at nodes shows a probability bootstrap. * – indicates GenBank sequences, with reference numbers, from the NCBI database

In order to reveal genetic differentiation between two species, ITS sequences were used for the construction of a phylogenetic tree (Fig. 4) together with two outgroup taxa.

The ITS-derived phylogenetic tree showed a clear separation of *Agriophyllum* species into two clades, i.e. *A. squarrosum* and *A. minus*. Two populations of *A. minus* and NCBI GenBank sequence had identical ITS sequences. In contrast, the population of *A. squarrosum* from western Kazakhstan and the GenBank sequence from China formed a clade separate from the populations collected in the southeastern region of Kazakhstan.

**Fig. 5:**
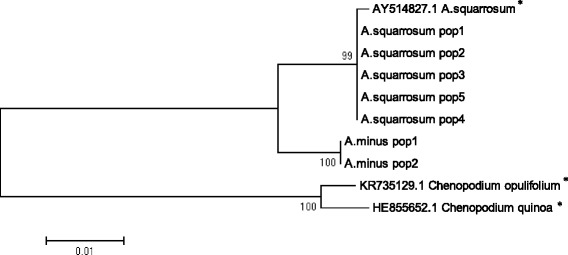
Maximum Likelihood phylogenetic tree resulted from analysis of the *mat*K sequences. The length of branches is based on Maximum Composite Likelihood analysis and numbers at nodes shows a probability bootstrap. * – indicates GenBank sequences, with reference numbers, from the NCBI database

In the *mat*K tree (Fig. 5) the *Agriophyllum* species were grouped into two sister clades. Despite the large geographic distances between the studied populations of *A. squarrosum* and *A. minus*, the samples within both species displayed no infra-specific variation.

## Discussion

Sand rice is a common plant of extreme desert conditions of Asia with its well-adapted morphology and physiology. Economic importance, nutritional and fodder value, stress tolerance, and its use for fixation of shifting sands are underlining the importance of this species for humankind [6]. In the last decade, several studies have increased our knowledge on sand rice, discussing its potential economical relevance [9]. However, the literature survey is suggesting that so far there were no attempts to study the genetic diversity of the genus *Agriophyllum* using molecular tools. Beside this, it is important to obtain basic facts related to the diversity of morphological traits of plants growing in different parts of the *Agriophyllum* distribution area.

In this study two different species, *A. squarrosum* and *A. minus,* were collected in two different regions of the Central Asia, separated by more than 1250 km distance. Samples from seven different populations were collected and studied using basic morphological characters and two DNA markers, ITS and *mat*K.

Domestication of *Agriophyllum* species as a food crop or pasture and fodder crops requires particular morphological traits. Both species studied here are the most common representatives of genus *Agriophyllum* in Kazakhstan. The results of our work suggested differences in morphological traits of *A. squarrosum* and *A. minus* in both regions. Morphometric analysis of key characters, such as leaf length, seed weight, and number of seeds per spike, clearly showed a difference between *A. squarrosum* and *A. minus* (Table 3).

The alignment of ITS and *mat*K sequences in the study showed that variation in ITS (3.6% of variability) was two times higher than in *mat*K (1.8% of variability), suggesting that ITS analysis is providing better resolution in comparison with the analysis using *mat*K as a chloroplast marker. The difference in sequence variability can be explained by lower mutation rate of the plastid genome [31] and by the mainly non-coding nature of the internal transcribed spacers region of rDNA. The ITS region can accumulate large amounts of mutations but with only small certain constraints due to its functional role, and, therefore, this marker is commonly used in phylogenetic analysis and DNA barcoding [32, 33].

Despite this difference in sequence variability, the topology of phylogenetic trees using both markers showed congruency in separation of *A. squarrosum* from *A. minus*. DNA analysis of all plants in populations revealed no hybrid specimens among collected samples of *A. squarrosum* and *A. minus*, and suggested that for these two sympatric species no gene flow occurs, at least not for the analyzed populations.

## Conclusions

Our study is the first attempt to analyze the taxonomic relationship and genetic differences of *A. squarrosum* and *A. minus*, which are annual plant species adapted to the mobile sand dunes in desert and semi-desert regions of Central Asia and China. Representatives of seven populations of *A. squarrosum* and *A. minus* were analyzed using two universally recognized plant DNA barcode markers, ITS and *mat*K*.* The results obtained suggested that variability of ITS is twice as high as in *mat*K sequences. However, the phylogenetic trees from both markers are congruent, showing clear separation of the two species. The ITS tree separated the samples from the two areas of Kazakhstan, suggesting a possibility of incorporation of this marker in a broad analysis of population genetics study of *A. squarrosum*. The results of morphological analysis confirmed differences between *A. squarrosum* and *A. minus* for key agronomic traits, such as leaf length, seed weight, and number of seeds per spike.
